# A mutation in CCDC91, Homo sapiens coiled-coil domain containing 91 protein, cause autosomal-dominant acrokeratoelastoidosis

**DOI:** 10.1038/s41431-024-01573-3

**Published:** 2024-04-16

**Authors:** Yunlu Zhu, Yun Bai, Wannian Yan, Ming Li, Fei Wu, Mingyuan Xu, Nanhui Wu, HongSong Ge, Yeqiang Liu

**Affiliations:** 1 Shanghai Skin Disease Hospital, School of Medicine, Tongji Medical University, Shanghai, China; 2 Department of Dermatology, Anhui Provincial Children’s Hospital, Hefei City, Anhui Province China; 3 Department of Dermatology, Children’s Hospital of Fudan University, Shanghai, 200032 China

**Keywords:** Genetics, Epigenetics

## Abstract

Acrokeratoelastoidosis (AKE) is a rare autosomal dominant hereditary skin disease characterized by small, round-oval, flat-topped keratotic papules on the palms, soles and dorsal aspect of hands or feet. The causative gene for AKE remains unidentified. This study aims to identify the causative gene of AKE and explore the underlying biological mechanisms. A large, three-generation Chinese family exhibiting classic AKE symptoms was identified. A genome-wide linkage analysis and whole-exome sequencing were employed to determine the causative gene. shRNA knockdown in human skin fibroblasts and CRISPR/Cas9 knockout in HEK293T cells were utilized to assess gene functions in the progression of elastic fiber biosynthesis. The linkage analysis identified a susceptibility region between rs7296765 to rs10784618 on chromosome 12. Whole-exome sequencing confirmed a splicing mutation of 1101 + 1 *G* > *A* in the *CCDC91* gene, resulting in exon 11 skipping and a subsequent 59-amino-acid-residue loss (residues L309-Q367del). Further functional analysis revealed distended Golgi cisternae, cytoplasmic vesicle accumulation, and lysosome presence. Immnunostaining of si-CCDC91-HSF cells demonstrated tropoelastin accumulation in the Golgi and abnormal extracellular aggregates. There are no significant changes in Fibrillin-1 microfibril assembly and lysyl oxidase activity. The findings strongly suggest that the protein product of the *CCDC91* gene plays a crucial role in elastin transport. This discovery enhances our understanding of *CCDC91*’s function and broadens the known pathogenic mechanisms of AKE.

## Introduction

Acrokeratoelastoidosis (AKE) (OMIM:101850) is an autosomal dominant hereditary skin disorder characterized by bilateral multiple hyperkeratotic papules primarily distributed on the lateral portion of the palmoplantar regions. It was initially described by Oswaldo Costa in 1953 [1, 2]. Clinically, the first lesions typically develop during childhood or early adulthood and progressively increase in size and number, eventually becoming grouped or forming plaques [3, 4].

The primary etiology of AKE remains elusive. Both sporadic and familial cases of autosomal dominant inheritance have been reported in the literature. Previous studies have suggested that light exposure and trauma may constitute risk factors for sporadic cases [5]. In 1983, Greiner et al. demonstrated a potential linkage to chromosome 2 based on genetic mapping of 21 affected members [6]. Although AKE was classified as type III punctate palmoplantar keratoderma (PPKP3) in 1996 [7], some pathogenesis elements of palmoplantar keratoderma, such as the *AAGAB* gene in type I palmoplantar keratoderma (PPKP1), have been found not to be involved in AKE [8]. Consequently, current research still has not identified the pathogenic gene of AKE.

In this study, we report the identification of a splicing mutation in the *CCDC91* gene (OMIM: 617366) within a Chinese family affected by AKE. Further functional analysis suggests that this gene may influence vesicle transport within the Golgi apparatus, thereby interfering with the synthesis and secretion of elastin.

## Materials and methods

### Subjects and clinical characteristics

**Fig. 1:**
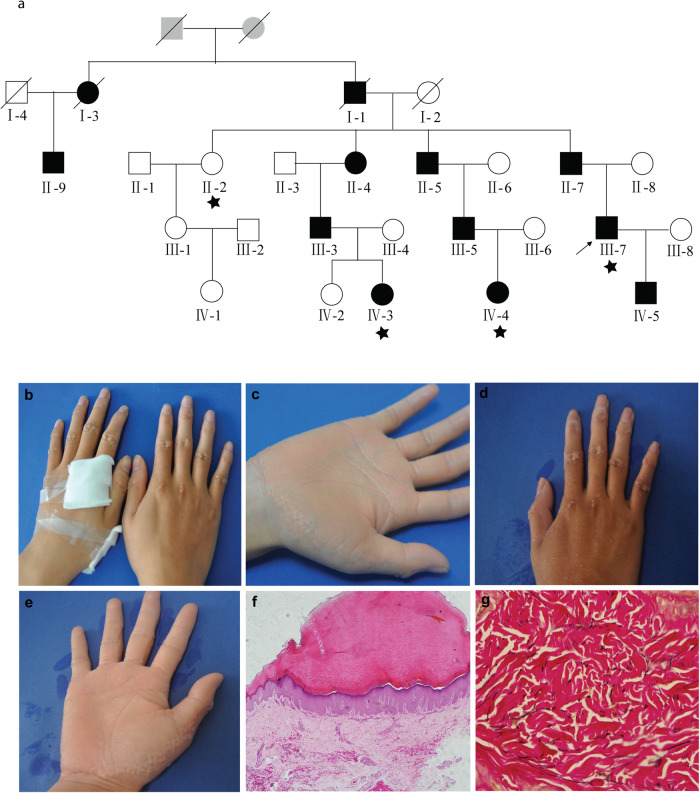
Family pedigree, clinical manifestations, and histological characteristics of individuals with AKE. **a** AKE family Pedigree. Circles and squares denote females and males, respectively. Deceased individuals are indicated by a diagonal slash. The proband is identified by an arrow and affected individuals are represented by black shading. ‘star’ within the pedigree indicates individuals who underwent whole-exome sequencing. **b** Clinical manifestations include symmetrical keratotic papules overlying the metacarpophalangeal and proximal interphalangeal joint protuberances in the proband. **c** Multiple, yellow-white, flat-topped papules predominantly distributed along the dosal palmar junction in the proband. **d, e** White, spongy appearance of the palm and dorsum of the hands after 5 min of immersion. **f** Histological findings reveal marked hyperkeratosis, a thickened granular layer, and acanthosis in the epidermis, along with sparse inflammatory cell perivascular infiltration in the superficial dermis. (Hematoxylin- eosin stain; original magnification ×40). **g** Fragmentation and diminution of elastic fibers in the reticular dermis (Verhoeff–van Gieson stain, original magnification ×400).

In this study, we recruited a three-generation AKE family from Jiangsu province, China, consisting of 22 living individuals, including ten males and twelve females. (Fig. 1a). All patients were diagnosed by experienced clinical dermatologists at the Shanghai Skin Disease Hospital based on typical clinical manifestations and histological features. The proband (III-7), a 28-year-old man, presented an 18-year history of multiple keratotic papules on his hands. The first small asymptomatic papule developed on his right hand and gradually increased in number. Further physical examination revealed multiple, flesh-colored, shiny papules symmetrically present along the margins of both hands, resembling a “paving stone” appearance (Fig. 1b, c). After immersion for 5 min, the rash on the palms and back of the hands became swollen and whitish (Fig. 1d, e). None of the patients had a history of hyperhidrosis, trauma, or excessive sun exposure. A skin-biopsy specimen from a papule, collected from the proband via skin biopsies, was stained with traditional hematoxylin and eosin (H&E) stain and Verhoeff-Van Gieson (VVG) stain. Histological examination revealed thick hyperkeratosis within focal cup-like depressions of the epidermis, hypergranulosis, slight acanthosis, and sparse perivascular lymphocytic infiltration in the superficial dermis (Fig. 1f). VVG stain revealed thick, curved, coarse, fragmented, and rarefied elastic fibers in the reticular dermis (Fig. 1g).

### DNA Isolation

Following informed consent, peripheral venous blood samples were collected from the proband and his family members. Genomic DNA was extracted from peripheral blood using the QIAamp DNA Blood Mini Kit (catalog number 51304, Qiagen, Valencia, CA, USA). The concentration and purity of the DNA were assessed utilizing a NanoDrop2000 Spectrophotometer (Thermo Scientific, Waltham, USA).

### Genotyping and linkage analysis

A comprehensive genome-wide SNP genotyping was executed employing the Illumina Human 660W-Quad_v1 BeadChips (Illumina, San Diego, CA), encompassing 655,214 SNP and copy number variation (CNV) probes. Ultimately, 6,886 markers were examined through multipoint parametric linkage analysis using the MERLIN program (version 1.1.2; Ann Arbor, MI, USA).

### Whole-exome sequencing

A total of 1 µg of DNA from the proband and three family members was employed to generate genomic DNA libraries, adhering to the protocols recommended by Illumina. Subsequently, Roche NimbleGen’s SeqCap EZ Human Exome Library v3.0 (64 Mb) was utilized for capture the genome’s exon regions. Following capture and PCR amplification, the libraries underwent sequencing via Illumina Hiseq 2000 Analyzers (Illumina, San Diego, USA). The remaining reads were aligned to the standard human genome reference (hg19) using Burrows-Wheeler analysis with default parameters [9]. Variants were sequentially filtered against dbSNP138, the 1000 Genomes project, and Hapmap 8.

### Sanger sequencing validation

All potential mutations identified through next-generation sequencing (NGS) were further confirmed via Sanger sequencing. The primer sequences are provided in Table S1. The PCR products were purified using the TIANgel Midi Purification Kit (TianGen, Beijing, China). The purified PCR products were examined with the 3730xl Capillary Sequencer (Applied Biosystems). Sequence comparisons and analyses were conducted using DNASTAR software.

### Reverse transcription-polymerase chain reaction (RT-PCR)

Peripheral blood mononuclear cells (PBMCs) were isolated via Ficoll-Uromiro cell density centrifugation. Subsequently, total RNA from the PBMCs was extracted using TRIzol Reagent (Invitrogen). Design primers around the splicing site (Table S1)and synthesize cDNA using the PPrimeScript^TM^II 1st Strand cDNA Synthesis Kit (TaKaRa Bio Inc, Tokyo, Japan). Finally, the PCR products of the proband and healthy individuals were sequenced.

### Cell culture

Human skin fibroblasts (HSF) were obtained from the Laboratory of Cell Biology at the Second Military Medical University (Shanghai, China). Cells were cultured in high-glucose Dulbecco’s Modified Eagle’s Medium (DMEM, Gibco) supplemented with 10% fetal bovine serum (FBS, BI) and 100 units of penicillin-streptomycin (Hyclone). Human embryonic kidney (HEK) 293 T cells were cultivated in the same medium with the addition of 10% FBS. All cell lines were maintained at 37 °C in a humidified atmosphere containing 5% CO_2_.

### Knockdown of CCDC91 gene in HSF

Four distinct short hairpin RNA (shRNA) sequences were designed based on the *CCDC91* reference sequence (Gene Bank Accession No. NM_018318.5) using online siRNA tools (Table S1). The target sequences were synthesized and cloned into an over-expressing lentiviral vector. All specific shRNAs were verified through sequencing.

The vectors were transfected into 293 T cells to generate packaged lentivirus, which was subsequently screened by adding 3 µg /ml puromycin. After determining the virus titer, the lentiviral stock was transfected into the HSF cell line. To assess the interference effect, *CCDC91* expression was detected using quantitative real-time PCR (Q-PCR) and western blot techniques. Q-PCR was performed using SYBR Premix Ex Taq^TM^ II (Takara Bio Inc., Tokyo, Japan) on a LightCycler 480 real-time platform (Roche, Basel, Switzerland). The primers are presented in Table S1, and GAPDH was measured as the house-keeping gene.

### CRISPR/Cas9 knockout of exon 11 in HEK293T

Based on the *CCDC91* gene sequence, two guide RNAs were designed to target exon 11 (Table S1). The sequences were validated using the CRISPR design tool (http://crispr.mit.edu/) and cloned into pSpCas9-2A-Puro (PX459) employing the BbsI enzyme (Thermo Fisher Scientific). The plasmid was cloned into *Escherichia coli* (DH5a) and subsequently extracted using a Miniprep kit (Qiagen).

HEK293T cells were transfected using Lipofectamine 2000 (Thermo Fisher Scientific). Following 8 h of transfection, cells were cultured in complete, fresh DMEM medium and screened by adding 3 µg /ml puromycin. Finally, the cDNA of the CCDC91 gene was sequenced to evaluate the knockout effect.

### Immunofluorescence analysis

To investigate the function of CCDC91, we assessed the structural impairment of the Golgi apparatus by performing immunofluorescence analysis in HSF cells with low CCDC91 expression and HEK293T cell which was knockout CCDC91 exon 11. Cells grown on glass coverslips were fixed in 4% formaldehyde for 10 min. After washing with PBS, cells were blocked for 30 min in 1% BSA, and subsequently incubated with primary antibodies against human CCDC91 (sc-514452, Santa Cruz) and GM130 (12480 T, Cell Signaling Technology) at 4 °C overnight. Different secondary antibodies were used for the two cell lines, anti-mouse IgG Alexa Fluor 488 (Invitrogen) and anti-rabbit IgG Alexa Fluor 568Cy^TM^3 (Invitrogen) in HSF and anti-mouse (IgG Alexa Fluor 488 CST) and anti-rabbit IgG Alexa Fluor 594Cy^TM^3 (CST) in HEK293T. DNA was stained by DAPI. Images were acquired and analyzed using laser scanning microscopy (LSM880, Carl Zeiss, Germany).

Furthermore, to investigate the influences of CCDC91 gene on elastic fiber, the expression and distribution of the human recombinant tropoelastin (TE), human aortic alpha elastin, and human fibrilin-1 were evaluated using immunofluorescence analysis in HSF cells with low CCDC91 expression. against TE (PR398, Elastin Products Company, Owensville, MO, USA), human aortic alpha elastin (PR533, EPC), human fibrilin-1 (NBP1-84722, NOVUS biologicals).

### Transmission electron microscope (TEM)

Cultured HSF and HEK293T cells were harvested and fixed in 0.1 mol/L phosphate buffer containing 2.5% glutaraldehyde at 4 °C overnight. Cells were then washed and post-fixed in 1% OsO_4_ buffer for 1 h at 4 °C. Following dehydration in a graded ethanol series, the samples were infiltrated with a Spurr resin and propylene oxide mixture for 24 h at room temperature. Finally, the samples were embedded in Spurr resin. Ultrathin sections were prepared using an EM UC6 ultra-microtome (Leica, Wetzlar, Germany) and stained with 3% uranium acetate and lead citrate at room temperature for 10 min. The samples were observed and imaged using a transmission electron microscope (JEM-1230, Japan) operating at 120 kV.

### Enzyme-linked immunosorbent assay (ELISA)

Culture medium was collected from fibroblasts after 10 days cultures. Insoluble elastin was quantified using the Human Elastin SimpleStep ELISA kit (ab239433) following the manufacturer’s instructions.

### Statistical and bioinformatics analysis

Results are presented as mean ± standard deviation (SD). Differences among groups were assessed using one-way ANOVA, with a *p-*value of less than 0.05 considered statistically significant. The three-dimensional structure model of the CCDC91 protein was generated using a combination of software applications, including FR-t5-M [10, 11] and I-TASSER [12]. The wild-type and mutant forms of CCDC91 were optimized using the side-chain packing program CIS-RR [13]. The structure models were visualized using PyMOL software (www.pymol.org/). Additionally, the impact of the deletion mutation(L309_Q367del) in CCDC91 was evaluated using the PROVEAN [14].

## Results

### Linkage analysis of the AKE Family

**Fig. 2:**
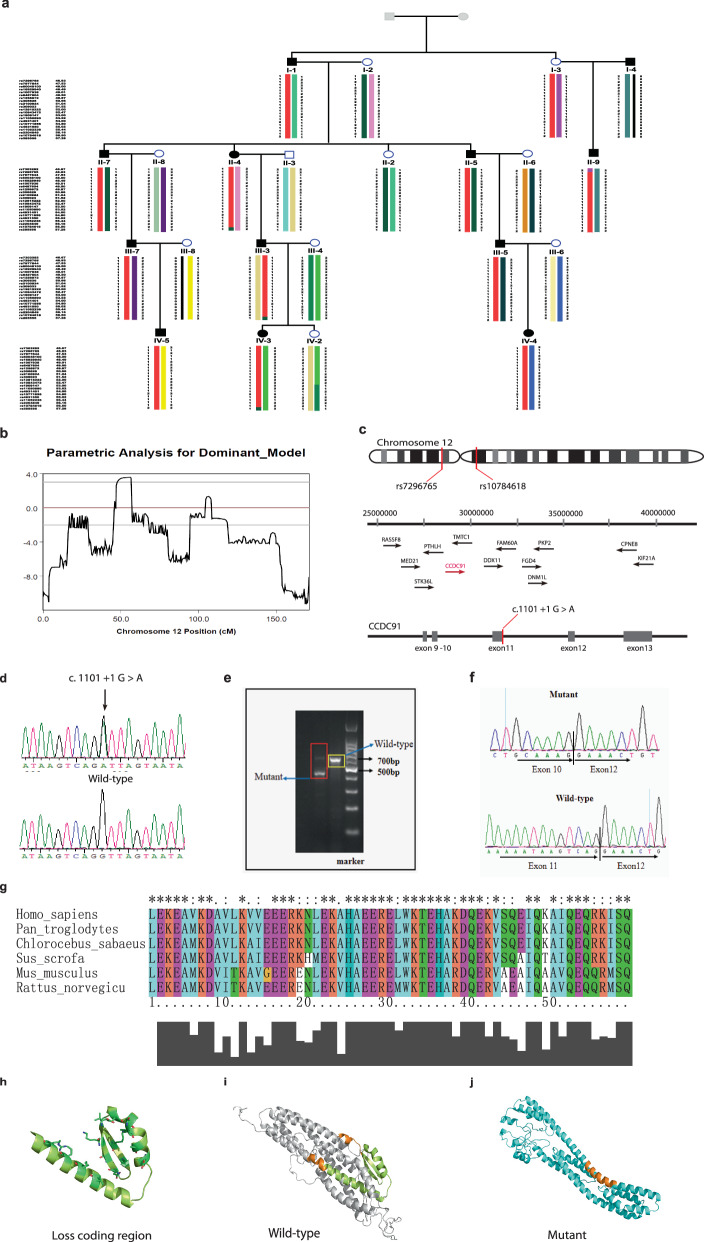
Identification of CCDC91 pathogenic genes and the prediction of the three-dimensional structure of CCDC91 protein. **a** Haplotype analysis of this family reveals a single chromosomal region on chromosome 12 (46.93–56.50 cM) shared by all affected family members, located between rs7296765 and rs10784618. Red bars indicate the shared chromosomal region among affected individuals. **b** Chromosome 12 multipoint LOD score analysis output, with maximal LOD score was 3.55. **c** Putative pathogenic genomic interval and location of the mutation on *CCDC91* gene (NM_018318.5). **d** Sanger sequencing revealed a heterozygous splice mutation (c.1101+1 G > A) in this family. **e** Agarose gel electrophoresis image displaying cDNA fragments from unaffected and affected individuals. “Mutant” represents for affected individuals, and “Wild-type” represents for healthy individuals. **f** Sanger sequencing of RT-PCR products reveals that the c.1101+1 G > A splice mutation leads to deletion of exon 11 from mature RNA. **g** Overview of the predicted structure. **h** The deletion mutation (L309_Q367del) is depicted in lemon yellow, with the residues EALVSAAK (E301-K308) and ETVKAAII (E368-I375), which are immediately adjacent to the mutation in the sequence, shown in orange. **i** The wild-type CCDC91 protein structure is displayed in gray. **j** The mutant CCDC91 protein structure is represented in cyan.

Owing to the current lack of reports on the genetic causes of AKE, we conducted genotype analysis on 17 individuals from this AKE family. Linkage analysis revealed chromosome 12 (46.93–56.50 cM), located between rs7296765 to rs10784618, as the susceptibility region due to the shared common haplotype among all affected individuals (Fig. 2a). The maximal LOD score was 3.55 (Fig. 2b, Table S3).

### Whole exome sequencing identified *CCDC91* mutations

We performed whole exome sequencing (WES) on four individuals from this family (Fig. 1a). We identified eight possible variants that appeared to be pathogenic (Table S2), and only the *CCDC91* gene located in the susceptibility region identified in the linkage analysis (Fig. 2c).

Further validation with Sanger sequencing confirmed a heterozygous splice mutation in *CCDC91*(NM_018318: exon11: c.1101+1 G > A), which was present in the affected individuals, but absent in unaffected individuals (Fig. 2d).

To determine the function of splicing mutations (c.1101+1 G > A), primers were designed in the mutation site area for RT-PCR. The results of electrophoresis showed that the healthy individual had only one cDNA fragment (606 bp), while patients exhibited two bands (606 bp and 429 bp) (Fig. 2e), suggesting that an abnormal cDNA fragment was generated by alternative splicing in *CCDC91*. Further Sanger sequencing indicated that c.1101+1 G > A mutation could result in the deletion of exon11 from mature RNA (Fig. 2f), which caused a 59-amino-acid-residue loss in the *ccdc91* protein (residues L309-Q367del).

### Bioinformatics prediction of structural changes in alternative splicing transcript

Due to the exon deletion observed in exon 11 of *CCDC91*, we assessed the evolutionary conservation of the missing amino acid residues and discovered that they were highly conserved among mammalian species (Fig. 2g). Further structural analysis revealed that the lost residues (L309_Q367del) form a stable motif comprising two alpha-helices opposing antiparallel beta-sheets (Fig. 2h). In comparison to the wild-type *ccdc91* protein (Fig. 2i), the mutant protein exhibits a narrower structure and a more concave surface, which may significantly impact its function (Fig. 2j). This result is also supported by the PROVEAN prediction of the deletion mutation (L309_Q367del), which is deleterious (−115.041).

### Abnormal Golgi apparatus in *ccdc91* knockdown of HSF and Exon 11 knockout in HEK293T

**Fig. 3:**
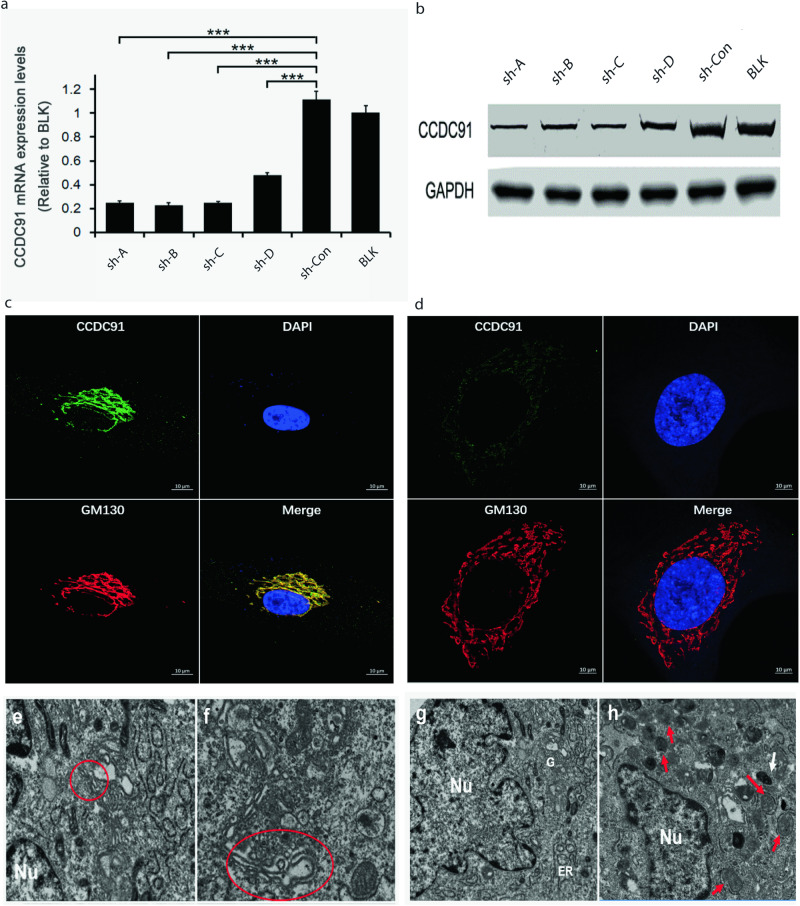
Evaluation of *CCDC91* knockdown effectiveness and its impact on Golgi apparatus structure. **a, b** Effectivency of *CCDC91* knockdown was assessed using quantitative real-time PCR and Western blot in human fibroblast cells. Error bars represent means ± SD. ***P < 0.001. Results indicated that the Sh-B group exhibited higher knockdown efficiency. **c** Immunolabelling of control HSF with antibodies against *ccdc91* (green) and GM130 (red) demonstrated significant co-localization of overlapping signals, suggesting subcellular localization of *ccdc91* in the Golgi apparatus. **d** Weak green signals and diffused red signals observed in the Sh-B group indicate successful *CCDC91* gene knock-down. Scale bar 10 μm. **e, f** Golgi body structure in the control and *CCDC91* knockdown group, respectively. In comparison to the control group, the Golgi apparatus in the *CCDC91* knockdown group appears more swollen and fractured into numerous small pieces. **g** The control group exhibits normal morphology of the nuclear (Nu), Golgi apparatus **g** and endoplasmic reticulum (ER). **h** Increased lysosomes (white arrows) and autophagosomes (red arrows) are observed in the *CCDC91* knockdown group.

To investigate whether the *CCDC91* gene affects the AKE phenotype, we introduced packaged shRNA-encoding lentivirus into HSF cells. A successful knockdown of *ccdc91* expression was observed via QPCR and Western blot (Fig. 3a, b). Considering that *ccdc91* is located in the Golgi apparatus, which is essential for elastin transportation, we performed immunofluorescence staining to detect *ccdc91* and Golgi marker GM130. As shown in Fig. 3c and 3d, significant co-localization of overlapping signals from GM130 (red) and *ccdc91* (green) was observed in both groups. Furthermore, we observed a dispersed, vesicular morphology of the Golgi apparatus in *CCDC91* knockdown cells compared to the negative control (Fig. 3f). Additional TEM analysis revealed that, compared to the negative control (Fig. 3e, g), the Golgi apparatus in the *CCDC91* knockdown group appeared more swollen and fragmented into numerous small pieces, suggesting that reduced *CCDC91* expression may lead to elastin transport disorder, causing its accumulation in the Golgi apparatus. Additionally, increased lysosomes and autophagosomes were observed in this group (Fig. 3f, h).

As the c.1101+1 G > A mutation results in the loss of exon 11 in the *CCDC91* gene, we employed the CRISPR/CAS9 system to knock out exon 11 in 293 T cell line (Fig. S1a, S1b). Compared to the negative control, we observed significant swelling of the Golgi apparatus in the CRISPR-CCDC91 293 T cell line (Fig. S1e, S1f).

### Abnormal tropoelastin deposition in *CCDC91* knockdown of HSF

**Fig. 4:**
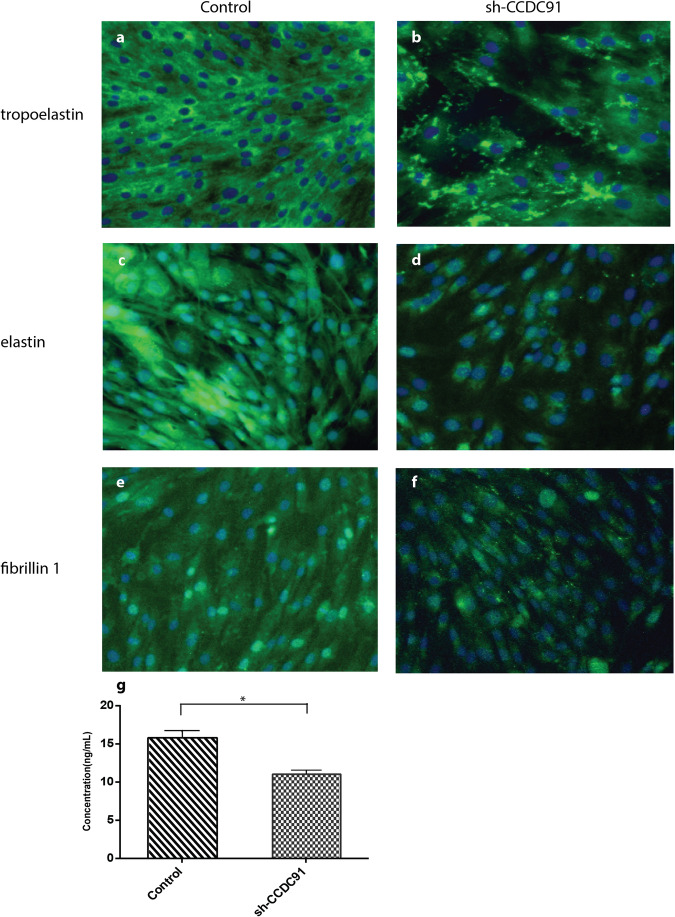
Immunofluorescence of elastic fiber-related proteins in *CCDC91* knockdown fibroblast. Cultured fibroblasts from control and *CCDC91* knockdown group were immunostained for tropoelastin **a, b**, elastin **c, d** and fibrillin 1 **e, f**. **g** Insoluble elastin from the culture media of control and *CCDC91* knockdown groups were quantified using ELISA. Bars represent means ± SEM, *< 0,05 (*t*-test).

Given that AKE is characterized by abnormal structure or quantities of elastic fibers, and tropoelastin, elastin, and fibrillin microfibril are essential components for elastic fiber biosynthesis. Compared to control the normal control group, Immunostaining for insoluble elastin revealed a reduction in si-CCDC91-HSF (Fig. 4c, d). An increased immunoreactivity from globular TE deposits was identified in si-CCDC91-HSF (Fig. 4a, b). No significant differences in fibrillin-1 were observed between si-CCDC91-HSF and the control fibroblasts (Fig. 4e, f). These results further suggested that *CCDC91* may affect the structure of elastic fibers by interfering with elastin transport, while not impacting fibrillin-1. A subsequent quantitative assessment of insoluble elastin by ELISA demonstrated that the synthesis of insoluble elastin in si-CCDC91-HSF fibroblasts was significantly decreased compared to the control group (Fig. 4g).

## Discussion

AKE is considered a hereditary skin disease with autosomal dominant transmission [15–17], although its genetic causes remain unclear. A genetic study involving 21 affected members has provided evidence for the genetic linkage of AKE to chromosome 2 [6]. Based on the histopathological characteristics of elastic fibers, one hypothesized pathogenesis may be related to a reduced number and impaired function of fibroblasts in the dermis [18]. A report has suggested that fragmented elastic fibres were not only observed in the lesional skin but also in normal-appearing, non-sun-exposed skin, indicating that the disorder could be considered a generalized defect of elastic tissue [4].

In the present study, we identified a novel splice mutation in the *CCDC91* gene from a Chinese AKE family using linkage analysis and WES. Further Sanger sequencing revealed that the mutation was present in affected individuals but absent in unaffected individuals, confirming that the *CCDC91* gene is one of the pathogenic genes of AKE.

The *CCDC91* gene is located on the short arm of chromosome 12 (12p11.22). It has 13 exons, with the first three exons subject to alternative splicing [19]. Although the splicing is highly variable at the 5-prime exons, other exons are conserved, encoding a protein composed of 441 amino acids with a predicted central coiled-coil domain. Immunofluorescence analysis demonstrated that *ccdc91* colocalized with GGA1, GGA2, and GGA3, which is a critical accessory protein that promotes transport of carrier vesicles between the Golgi and lysosomes [20]. Although the function of ccdc91 is not well understood, some genome-wide association studies (GWAS) studies have reported that CCDC91 polymorphism may be associated with lung function and ossification of the posterior longitudinal ligament [21, 22]. A meta-analysis of 17 GWAS studies identified sentinel variants in or near the *CCDC91* gene associated with forced vital capacity (FVC), a key criterion for the diagnosing restrictive lung diseases such as pulmonary fibrosis [21]. Further expression analysis indicated that the *CCDC91* gene was over-expressed in lung tissue and was relevant to lung function in a DNase-seq experiment. These results suggest that the *CCDC91* gene may be an important regulator of elastic fiber homeostasis.

Previous studies have suggested that keratotic papules may be caused by over-production of filaggrin [23, 24]. while disturbances in elastic fiber have been observed in some AKE cases [25]. Elastin, the most abundant protein in elastic fibres, constitutes nearly 90% of the mature structure [26]. It is encoded by the ELN gene and initially synthesized as a soluble precursor tropoelastin (TE) polypeptide on the ribosome. Following synthesis, the secreted TE polypeptide is transported to the endoplasmic reticulum for preliminary processing and then to the Golgi apparatus for further modification [27]. Finally, soluble TE is secreted from the Golgi apparatus into the extracellular matrix, where it aggregates to form spherical elastin [28, 29]. During this assembly process, the *CCDC91* gene, located in the Golgi, may experience mutations that interfere with tropoelastin transport, leading to tropoelastin accumulation within the Golgi and significantly reduced deposition of mature elastin. In this study, we utilized a CCDC91 knockdown cell line and a 293 T cell with exon 11 of *CCDC91* knockout, observing a significant swelling of the Golgi apparatus, increased lysosomes, and accumulation of vesicles in the cytoplasm. These findings further support our hypothesis that *CCDC91* gene mutations may affect vesicle transport within the Golgi apparatus and limit the synthesis and secretion of elastin.

Clinically, AKE is characterized by multiple, firm, round-to-oval, flesh-colored or yellowish keratotic papules along the lateral or medial borders of hands or feet, typically distributed in a bilateral and symmetrical pattern. However, some unilateral cases have been reported [30–32], which have been attributed to genetic mosaicism. In this study, we observed multiple, flesh-colored, shiny papules along the margins of both hands in affected individuals, suggesting that the pathogenic mutation may have originated from germline cells. After immersing the hands for five minutes, lesions appeared swollen and whitish. Similar clinical phenotypes have been described in aquagenic palmoplantar keratoderma (APK), characterized by translucent or whitish papules on the palms and soles that develop after brief water exposure [33, 34]. Although several studies have linked APK with cystic fibrosis transmembrane conductance regulator (*CFTR*) dysfunction [35, 36], we did not identify any *CFTR* gene mutations in our study. Interestingly, a previous report described a patient with concurrent AKE and APK appearing, yet without *CFTR* gene mutations [37]. These findings further suggest that AKE pathogenesis may differ from APK, and the thick hyperkeratosis induced by AKE potentially altering ion or protein channels of the eccrine ducts.

Although familial and sporadic AKE have been reported, the underlying pathogenic genes of AKE are still unknown. Our probands and their affected family members exhibit characteristic clinical and histological features of AKE. In this study, we also collected two sporadic cases and detected the causative gene by NGS. However, we did not find any genetic variants in the CCDC91 (Table S4). Therefore, we inferred that the pathogenic mechanism of hereditary AKE may be different from sporadic cases. Further genetic studies are warranted to test this speculation.

In summary, we investigated a Chinese family presenting with characteristic AKE lesions on the palms, soles and dorsal aspect of hands or feet. Through linkage analysis and deep sequencing, we identified a causative mutation in the CCDC91 gene. Our study further suggests that the ccdc91 protein may function as a regulator, enhancing the vesicular transport of elastin from the Golgi apparatus to the extracellular matrix. Its dysfunction may lead to abnormalities in elastic fibers, potentially contributing to the typical lesions observed in AKE patients.

### Supplementary information

Figure S1

TableS1-S3

TableS4

Abnormal Golgi apparatus in CCDC91 Ex11 knockout HEK293T cell.

## Data Availability

The datasets generated during the current study are available from the corresponding author.
