# Supplementary figures and images for: A mutation in CCDC91, Homo sapiens coiled-coil domain containing 91 protein, cause autosomal-dominant acrokeratoelastoidosis
Source: Eur J Hum Genet. 2024 Apr 16;32(6):647–55. doi: 10.1038/s41431-024-01573-3 (PMC11153616; doi:10.1038/s41431-024-01573-3)

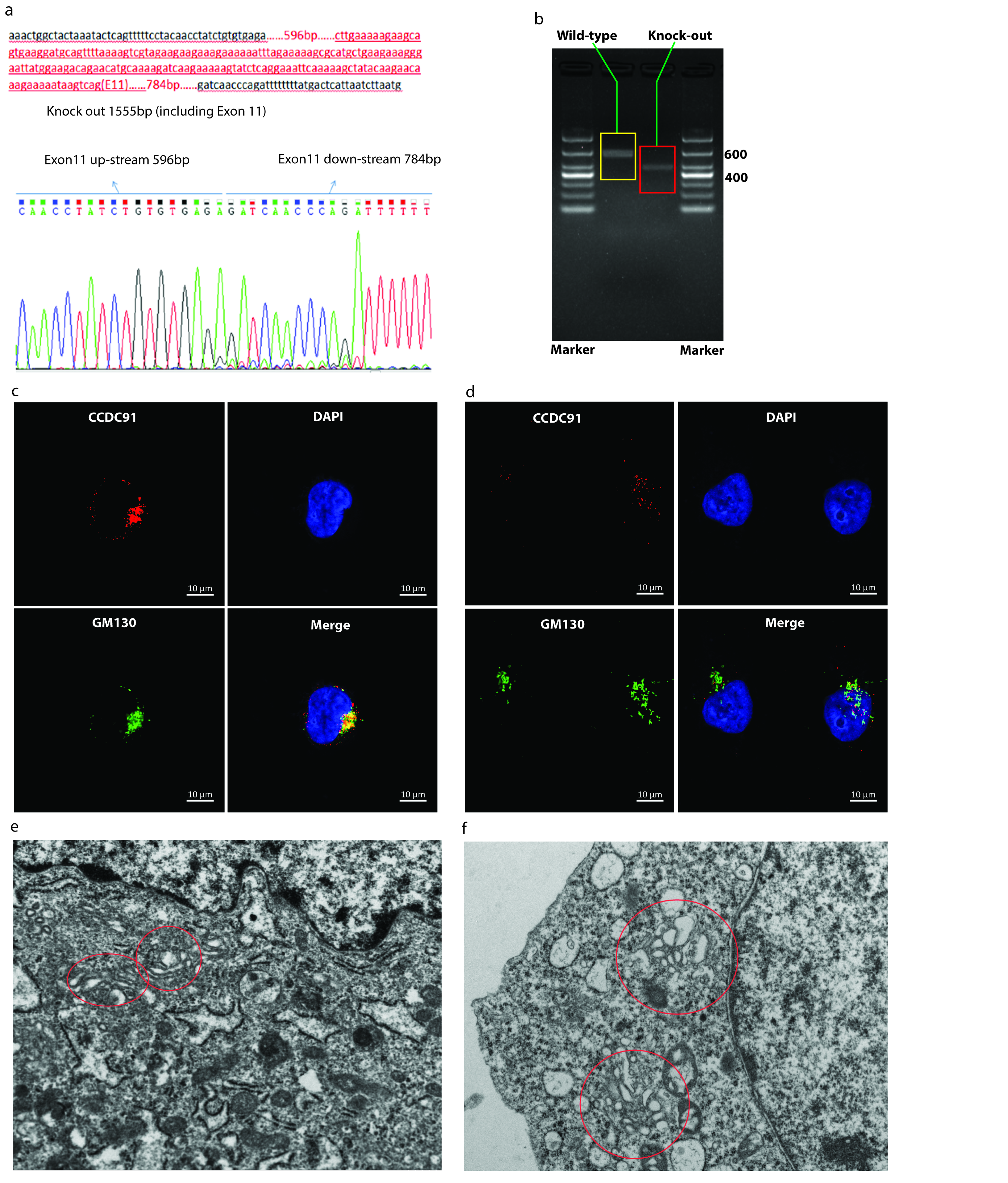

Supplement: Supplementary file 1 — Figure S1 [file 41431_2024_1573_MOESM1_ESM.tif]
