# Supplementary material for: A mutation in CCDC91, Homo sapiens coiled-coil domain containing 91 protein, cause autosomal-dominant acrokeratoelastoidosis
Source: Eur J Hum Genet. 2024 Apr 16;32(6):647–55. doi: 10.1038/s41431-024-01573-3 (PMC11153616; doi:10.1038/s41431-024-01573-3)
Supplement: Supplementary file 2 — TableS1-S3 [file 41431_2024_1573_MOESM2_ESM.docx]

**SupplementaryTable2 Identified potential pathogenic mutations of AKE family in this study.**

| Gene |  | Refseq NM | Nucleotide change | Amino acid change | Variant type | 1000G (All) | SNP138 | Polyphen2 | SIFT | Control |
| --- | --- | --- | --- | --- | --- | --- | --- | --- | --- | --- |
| OVCH2 |  | NM_19818 | c.G770A | p. G257D | Heterozygous | 0.0297524 | rs61759818 | D | D | 0/600 |
| CCDC91 |  | NM_018318 | c.1101+1G>A | \ | Heterozygous | 0.000599042 | rs201400994 | \ | \ | 0/600 |
| CELSR3 |  | NM_001407 | c.G469C | p. A157P | Heterozygous | 0.00219649 | rs3733085 | B | D | 0/600 |
| TRAIP |  | NM_005879 | c.G470A | p. R157H | Heterozygous | 0.00199681 | rs143366962 | B | T | 0/600 |
| CD180 |  | NM_005582 | c.A620G | p. N207S | Heterozygous | 0.00119808 | rs142324076 | B | T | 0/600 |
| OTP |  | NM_032109 | c.C212T | p. A71V | Heterozygous | 0.000599042 | rs185198254 | B | T | 0/600 |
| ERAP2 |  | NM_022350 | c.A1219G | p. T407A | Heterozygous | 0.000599042 | rs138646616 | B | T | 0/600 |
| RIOK2 |  | NM_018343 | c.G418A | p. D140N | Heterozygous | 0.000399 | rs144410790 | D | D | 0/600 |

**Key:** 1000G, 1000 Genomes Project;D (Polyphen2), probably damaging; B (Polyphen2): benign; T(SIFT), tolerated; D (SIFT), deleterious.

**Supplementary Table 2**Primer sequence for Sanger sequencing of candidate genes

**Table S1 Primer sequences in present study**

| Primer | Sequences（5’ to 3’） |
| --- | --- |
| **Sanger Sequence validation** |  |
| CCDC91-Fw | GTTTGAAATCTGCTACTCCACCCT |
| CCDC91-Rv | TGCATGTAGGTCATGCCTGTTG |
| TRAIP-Fw | GGGGTTTGGGAGTACATGGTTA |
| TRAIP-Rv | GACAGGGAGGAATGTGGCAAGG |
| RIOK2-Fw | ACACTTCTGGTCCGAAGGTTTT |
| RIOK2-Rv | AGCCCAAATAATCAACAGAGTT |
| ERAP2-Fw | AGAAGGCAAGGAAATCAGTAGAAG |
| ERAP2-Rv | GTAGCCATGAGCATTCCTGTGC |
| OTP-Fw | CCCCTTGCCTCCTCAGGTATGA |
| OTP-Rv | CTCGGTCAGCCCGATACGCAG |
| CD180-Fw | ATATTCATGGCAGAAACATCGC |
| CD180-Rv | CACTGAAATGTGGTGGATGAGA |
| CELSR3-Fw | GCATATCGGTGGCGGAGCCTTAG |
| CELSR3-Rv | TGTCCTCGCCGTGCGTGGTGCTGAA |
| OCVH2-Fw | AGGGAGGTGGGATTGTTGGGTT |
| OCVH2-Rv | GAGGTAGAGCCTGTTGGTTCAG |
| **CCDC91 RT-PCR** |  |
| CCDC91-As-Fw | GCAATTGAGAAACAGGCACAC |
| CCDC91-As-Rv | AATGTCAACTGGTTCCGTAGCT |
| **sh-RNA sequences** |  |
| CCDC91-A | GAAGAGCAGAAACGAAGTGAA |
| CCDC91-B | CAACATCTCCTGCTATTCCTT |
| CCDC91-C | GAGTCCATCTTTCACCATCTT |
| CCDC91-D | TAGCCTGTGGGAGTCTATTAT |
| Negative control | CCTAAGGTTAAGTCGCCCTCG |
| **Q-PCR** |  |
| CCDC91-Ex-Fw | GGCTGCGGAGACTTTTGATG |
| CCDC91-Ex-Rv | TGGTGAAAGATGGACTCCAGATA |
| GAPDH-Ex-Fw | ACAACTTTGGTATCGTGGAAGG |
| GAPDH-Ex-Rv | GCCATCACGCCACAGTTTC |
| **CRISPR/Cas9** |  |
| sgB1-F | CACCGCAACCTATCTGTGTGAGACT |
| sgB1-R | AAACAGTCTCACACAGATAGGTTGC |
| sgB2-F | CACCGAAAATCTGGGTTGATCTGAA |
| sgB2-R | AAACTTCAGATCAACCCAGATTTTC |

**SupplementaryTable3 multipoint LOD score of the susceptibility region.**

| SNP ID | Location | LOD |
| --- | --- | --- |
| rs7296765 | 25549022 | 2.6259 |
| rs7977844 | 25811125 | 2.9915 |
| rs60048100 | 26004016 | 3.1362 |
| rs16929940 | 26195527 | 3.2489 |
| rs1007938 | 26802549 | 3.3368 |
| rs6487604 | 27524824 | 3.3955 |
| rs1256972 | 27604435 | 3.4333 |
| rs306626 | 27668729 | 3.4714 |
| rs2100924 | 28493731 | 3.4898 |
| rs309023 | 28968916 | 3.5119 |
| rs12815222 | 29499578 | 3.5252 |
| rs10843472 | 29820426 | 3.5317 |
| rs1909147 | 30172287 | 3.5376 |
| rs11050890 | 30568174 | 3.5436 |
| rs4931401 | 31133664 | 3.5466 |
| rs10771856 | 31759101 | 3.5533 |
| rs4931550 | 31891056 | 3.5554 |
