# Supplementary material for: A mutation in CCDC91, Homo sapiens coiled-coil domain containing 91 protein, cause autosomal-dominant acrokeratoelastoidosis
Source: Eur J Hum Genet. 2024 Apr 16;32(6):647–55. doi: 10.1038/s41431-024-01573-3 (PMC11153616; doi:10.1038/s41431-024-01573-3)
Supplement: Supplementary file 4 — Abnormal Golgi apparatus in CCDC91 Ex11 knockout HEK293T cell. [file 41431_2024_1573_MOESM4_ESM.docx]

**Supplementary Figure 1. Abnormal Golgi apparatus in *CCDC91* Ex11 knockout HEK293T cell. (A-B)** The design of CRISPR/Cas9 knockout exon 11 in HEK293T cell, followed by agarose electrophoresis and Sanger sequencing to validate knockout efficiency. **(C)** Immunolabelling of control HEK293T cells with antibodies against *CCDC91* (red) and GM130 (green) reveals significant co-localization of overlapping signals. **(D)** Weak red signals and diffused green signals are observed in the Ex11 knockout HEK293T cell. Scale bar 10 μm. **(E-F)** Transmission electron microscope images of the Golgi apparatus in the control and *CCDC91* Ex11 knockout group, respectively. Compared to the control group, the Golgi apparatus in *CCDC91* Ex11 knockout group appears more swollen and fractured into numerous small fragments.
